# Combination of Suicide and Cytokine Gene Therapies as Surgery Adjuvant for Canine Mammary Carcinoma

**DOI:** 10.3390/vetsci5030070

**Published:** 2018-08-03

**Authors:** Liliana M. E. Finocchiaro, Agustina I. M. Spector, Lucrecia Agnetti, M. Florencia Arbe, Gerardo C. Glikin

**Affiliations:** Unidad de Transferencia Genética, Instituto de Oncología “Ángel H. Roffo”, Universidad de Buenos Aires, Av. San Martín 5481, 1417 Buenos Aires, Argentina; finolili@hotmail.com (L.M.E.F.); agustina.spector@gmail.com (A.I.M.S.); agnettilucrecia@gmail.com (L.A.); florenciaarbe@hotmail.com (M.F.A.)

**Keywords:** IL-2, GM-CSF, HSV-tk, IFN-β, canine mammary carcinoma, cancer vaccine, lipofection, DMRIE, gene therapy

## Abstract

The incidence of canine mammary carcinoma varies with age, breed, and spay status, being among the main tumors appearing in intact female dogs. Thirty-six canine mammary carcinoma patients received injections of canine interferon-β (cIFN-β) and HSV-thymidine kinase/ganciclovir (HSV-tk/GCV) carrying lipoplexes, into the tumor bed, immediately after surgery. Next, they started periodic subcutaneous injections of lipoplexes carrying a human granulocyte-macrophage colony stimulating factor and interleukin-2 mixed with allogeneic mammary carcinoma extracts. This combined strategy was safe and well tolerated. In addition, only two out of 26 patients treated with complete surgery developed a local relapse, and 0 out of 29 stage II and III patients displayed distant metastases, suggesting both local and systemic antitumor activities. The most encouraging result was the long survival times: 22 > 1 year (where 13 > 2 and 4 > 3 years), while maintaining a good quality of life. The preliminary results in five patients presenting with local disease, an additional HSV-tk/GCV plus cIFN-β gene treatment induced local antitumor activity, evidenced by four objective responses (one complete, three partial) and one stable disease. This successful outcome supports further studies to validate this approach not only for canine veterinary patients, but also for translation to human patients.

## 1. Introduction

Canine mammary carcinomas (CMCs) are among the most prevalent tumors in intact female dogs [1]. Even though an ovariectomy is an efficient preventive action, this is not a common practice in every geographical region. Among the CMCs, there are a variety of subtypes with different histologies [2]. Because of the similarities with human breast carcinoma, CMCs have been proposed as models for studying human carcinogenesis [3] and prognostic factors [4,5]. Lately, there were important advances in the understanding of the clinically relevant genetic and molecular pathways involved, driving cell proliferation, apoptosis and DNA repair, metastasis, and the interactions of tumor cells with other non-tumor cells in CMCs [6,7,8].

CMCs have less treatment alternatives in comparison with human breast cancer; many new therapies used for human patients were not validated for companion animals, and most of them would be too expensive and therefore prohibitive for owners. Therefore, in the veterinary oncology context, the development of new simple and affordable approaches is compelling. Along this way, cancer immunogene therapy is an especially suitable and fast expanding field that was reasonably explored for malignant melanoma and other kinds of canine spontaneous tumors [9,10]. It is worth mentioning that three small trials of immunogene therapy for mammary carcinoma have been reported. Two of them used IL-12 electrogene therapy [11,12] and the another used a p62 DNA vaccine [13].

Our laboratory has developed efficiently combined gene therapies as surgery adjuvants for canine melanoma [14], soft tissue sarcomas [15], and osteosarcoma [16]. The treatments combined the local antiproliferative activity of the herpes simplex virus thymidine kinase (HSV*tk*) suicide gene and canine interferon-β (cIFNβ) gene therapy with the systemic action generated by a subcutaneous vaccine made from formolized tumor extracts and slow cytokine releasing biological systems. The lipoplexes carrying human interleukin-2 (hIL-2) and human granulocyte-macrophage colony-stimulating factor (hGM-CSF) were effective as immune enhancers with the canine melanoma vaccine, and their components proved to be especially suitable for long term storage and delivery [14]. Taking into account our previous experience with immunogene therapy, we are presenting a similar approach for treating CMCs, in a new study testing the feasibility and safety of the combination of local suicide plus interferon-β, and a cytokine enhanced tumor vaccine.

## 2. Materials and Methods

### 2.1. Plasmids

The same psCMV plasmid backbone was used as a carrier of all of the genes. Herpes simplex thymidine kinase (psCMV*tk*), canine interferon-β (psCMV*cIFNβ*), human interleukin-2 (psCMV*hIL2*), and human granulocyte-macrophage colony-stimulating factor (psCMV*hGM-CSF*) genes were subcloned, amplified, purified, and diluted to a final concentration of 2.0 mg/mL in sterile phosphate-buffered saline, as described [14].

### 2.2. Liposomes Preparation and Local Lipoplexes Injection

The liposomes were made as previously reported [14]. They were composed of equimolar amounts of DMRIE (1,2-dimyristyl oxypropyl-3-dimethyl-hydroxyethylammonium bromide) and DOPE (1,2-dioleoyl-sn-glycero-3-phosphatidyl ethanolamine). The lipoplexes were assembled by mixing liposomes and plasmid DNAs (1:2 *v*:*v*) at room temperature for 10 min. Subsequently, 5 mg ganciclovir (GCV)/mg DNA was added and the mixture was injected intra- and/or peri-tumorally into multiple sites, as described in Section 2.5.

### 2.3. Tumor Vaccines Preparation

Surgically removed tumors were processed as previously described [17]. Aliquots of 0.05–0.10 mL of insoluble pellets of homogenized tumors were stored at −80 °C until used. No microbial cultivable contamination was found in the tumor preparations. The analyses for endotoxin presence (≤1 EU /μg protein) were performed using the Limulus Amebocyte Lysate assay. Autologous or allogeneic tumor cells prepared in 0.25 mL were mixed with 0.75 mL of lipoplexes carrying 0.5 mg psCMV*hIL2* plus 0.5 mg psCMV*hGM-CSF*, just before subcutaneous injection.

### 2.4. Patients

Inclusion criteria: Female dogs with a confirmed histopathological diagnostic of malignant mammary carcinoma and that were free of severe underlying systemic illnesses were evaluated for being entered into the study. There were no restrictions on the disease stage or burden. The tumors were staged according to the World Health Organization (WHO), as previously described [17], as follows: stage II, for primary tumors from 2 to 4 cm diameter with negative proximal lymph nodes; stage III, for tumors ≥4 cm diameter without metastasis or any size of primary tumor with lymph node metastasis; or stage IV, for distant metastatic disease. The staging methods included physical examination, thoracic radiography, abdominal echography, complete blood count, serum biochemistry profile, urinalysis, and coagulation profile. All of the patients entered the study with a modified Eastern Cooperative Oncology Group (ECOG) performance status of <2 (normal activity or decreased activity from pre-disease status), as previously defined [18]. Exclusion criteria: alkaline phosphatase ≥3× normal; hepatic transaminases ≥3× normal; total bilirubin ≥2× normal; creatinine ≥2× normal; <2000 neutrophils/μL; <100,000 platelets/μL; hematocrit <25%; and evidence of preexisting non controlled, non tumor-related cardiovascular, pulmonary, or immune disease. Neither chemotherapy nor any other potentially antitumor or immunosuppressive medication was administered to the dogs in the previous four weeks or during the study. Standard antibiotics, non steroid anti-inflammatory, and/or analgesic medication were used when needed. All of the dogs’ owners signed the corresponding written informed consent for this experimental treatment. The surgery procedures and clinical follow-up were performed by qualified attending veterinary professionals, following the laws and regulations of our country (Argentina). All of the scientific and ethical issues related to the veterinary clinical trial were evaluated and approved by the appropriate committee of the granting agency (ANPCYT, Buenos Aires, Argentina).

### 2.5. Study Design and Treatment

Three veterinary care centers recruited patients and took part in this prospective study. At the time of surgery, the patients were assigned, taking into account whether they were subjected to complete (CS) or partial (cytoreductive) surgery (PS).

**Figure 1:**
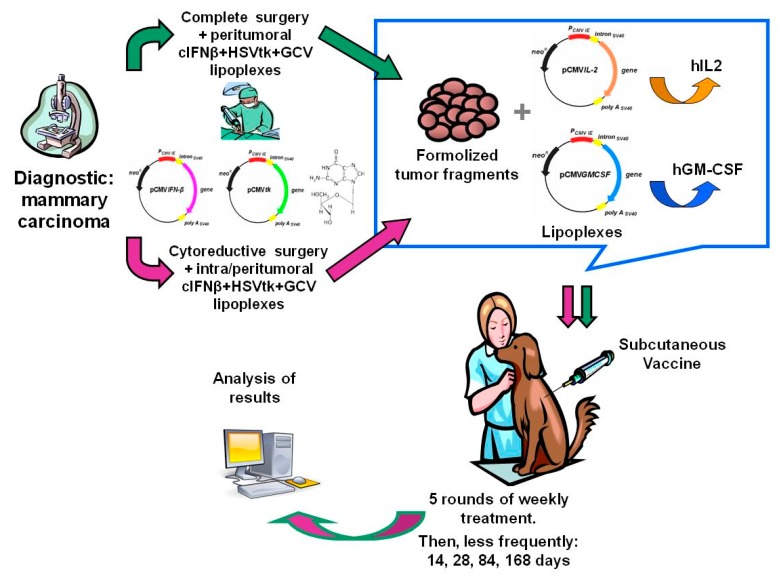
Diagram of the combined gene therapy as a surgery adjuvant.

**Figure 2:**
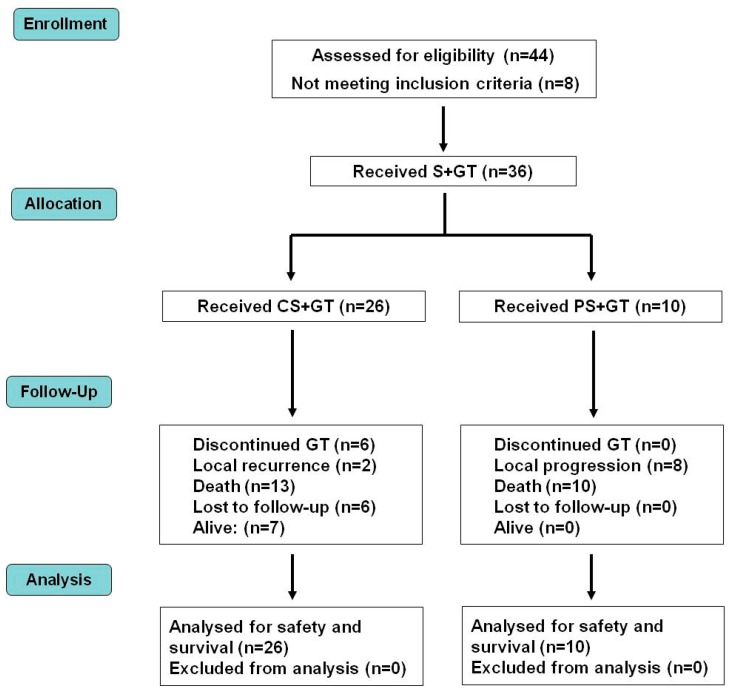
Distribution of patients in a CONSORT diagram. CS—complete surgery; GT—gene therapy; PS—partial surgery; S—surgery.

Figure 1 and Figure 2 display the general scheme of the treatment and the CONSORT chart, respectively [19]. The patients getting the combined treatment (*n* = 36) were subjected to partial (PS, *n* = 10) or complete surgery (removal of all detectable tumor burden followed by confirmation of clean surgical margins in the histopathological report) (CS, *n* = 26), as described [14]. The surgical margin of the cavity produced by the tumor removal was injected with cIFNβ and suicide gene carrying lipoplexes (1 to 4 mg DNA of each) co-delivered with GCV (5–20 mg), according to the tumor bed (about 0.1 mg DNA/cm^2^ of surgical margin). The injections were uniformly spread at multiple locations in the surrounding areas and/or in the remaining tumor tissue. Starting at surgery, the patients were treated once a week for five weeks with a subcutaneous vaccine composed by allogeneic formolized tumor cell extracts (containing about 0.05–0.10 mL of insoluble pellet) and lipoplexes carrying the genes of hIL-2 and hGM-CSF (0.5 mg DNA of each cytokine). After that, the patients received a subcutaneous vaccine with decreasing frequency five times (5×) every other week, three times monthly, three times monthly, every three months, and lastly, every six months until relapse or death.

In a parallel exploratory group, at a local recurrence (*n* = 4) or local disease progression (*n =* 1), patients were subjected to weekly intratumoral injections of cIFNβ and suicide gene carrying lipoplexes co-delivered with GCV, as described above. They also restarted the weekly subcutaneous vaccine schedule. The follow-up lasted until the patients’ death or the end of the study. For the sake of simplicity, a local relapse was considered as a recurrence in the same place, invasion of surrounding tissue, and/or regional metastasis (including very proximal lymph nodes). As reported in a previous study [15], the tumor responses were evaluated following WHO criteria.

As previously described [17], the periodic clinical evaluations were performed every treatment day and were completed by a monthly or bimonthly clinical laboratory analysis. Thoracic radiographs and abdominal echographs were done before treatment, and every month, or trimester, according to the patients’ response, and at longer intervals (six months) in long-term surviving animals. The patients subjected to a second local treatment also restarted the (i) periodic clinical evaluations every treatment day, and the (ii) thoracic radiographies and abdominal echographies every month.

The quality of life was assessed by a questionnaire completed by the owner before every treatment session, as described [17].

The following endpoints were determined: overall survival (OS), survival to CMC (MS), recurrence-free survival (RFS), metastasis-free survival (MFS), and disease-free survival (DFS). OS and MS were defined as the periods of time between first treatment day and death due to any cause or to CMC, respectively. If death was not observed during the study, the data on OS and MS were censored at the last date the patient was known to be alive. RFS, MFS, and DFS were defined as the interval from the initiation of the treatment to recurrence, metastasis, or the first detection of any of them, respectively. The dogs were censored from the analysis if they were disease-free at the time of the last follow-up or lost to follow-up.

Statistical studies of survival data were made by a Kaplan-Meier analysis, where y = the curves were compared by a Log-rank test, while response data were analyzed by 2-tail Fisher’s Exact test.

## 3. Results and Discussion

A detailed description of the treatment scheme appears in the Materials and Methods section, in Figure 1, and a CONSORT chart [19] of the trial is shown in Figure 2.

The patients’ median age at recruitment was 12 (5–15) years (Table 1), and the mean age 11.28 ± 0.35 years. The main breeds were the Cocker spaniel (19.4%), German shepherd (11.1%), Rottweiler (5.6%), and Schnauzer (5.6). The majority of the patients belonged to mixed breeds (33.3%) and other diverse breeds (25.0%).

The histological types found in this study were considerably diverse and a proportion of the patients presented with an advanced clinical staging, which negatively impacts the overall survival. The main types were tubulopapillary (22.2%), solid (19.4%), tubular (16.7%), ductal (11.1%), complex (8.3%), anaplastic (5.6%), and other diverse types (16.7%). Every patient was staged as follows: Most of them were at stage III (50%) or stage II (31%). Nearly 19% of the patients had lung metastases (stage IV) when incorporated to the trial.

**Figure 3:**
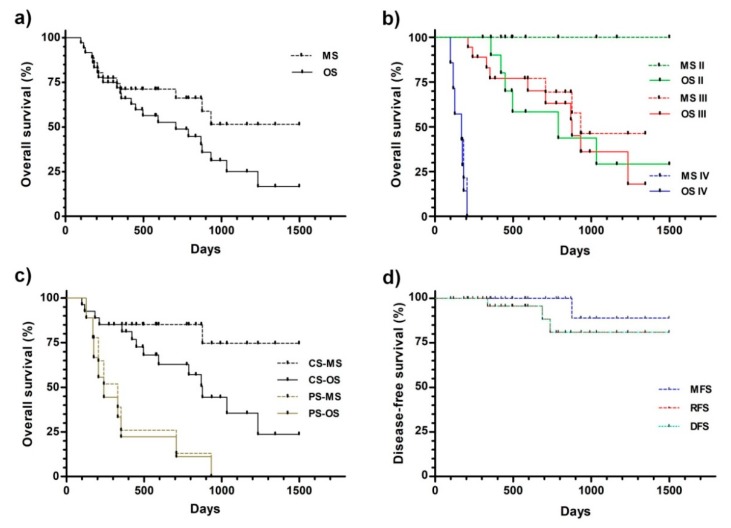
Kaplan–Meier analysis of the overall (**a**–**c**) and disease-free (**d**) survival. (**a**) All of the patients were considered as a single group. (**b**) The patients were grouped by the initial disease stage. (**c**) The patients subjected to partial (PS) and complete (CS) surgery were differentially grouped. (**d**)Disease-free survival. The patients were treated and follow-ups were performed, as described in the Materials and Methods. Abbreviations: OS—overall survival to any cause (death related or unrelated to CMC); MS—overall survival to CMC (death related to CMC); DFS—disease-free survival; RFS—recurrence-free survival; MFS—metastasis-free survival.

Figure 3 displays the Kaplan–Meier plots obtained from survival data. The median overall survival of the whole group was 708 days, but if only the deaths related to CMC were considered, it was >1498 (Figure 3a and Table 2). Nevertheless, the survival curves were not significantly different.

The median survivals appeared to be related to the CMC stage at the beginning of the treatment (Figure 3b and Table 2). For both stages II and III, the median survivals were significantly higher than the corresponding stage IV values.

The disease stage at the beginning of the treatment significantly impacted the quality of the surgical procedure. Partial surgery was performed only when necessary, in the patients with a poorer prognosis at higher disease stages, stages III (six patients) and IV (five patients). Therefore, the overall median overall survival of the latest group was significantly lower compared with the complete surgery group (241 vs. 876 days, Figure 3c and Table 3, respectively). This difference was higher when only the deaths related to CMC were considered (330 vs. >1498 days).

The metastasis- (MFS), recurrence- (RFS), and disease-free (DFS) survivals did not reach the median during the study, being >1498 days for the three parameters (Figure 3d and Table 3). It is noteworthy that none of the stage II and III patients developed distant metastases during or after treatment. Only one (out of 11) stage II, one stage III (out of 12), and 0 stage IV (out of 3) patients that were subjected to complete surgery displayed local recurrence.

It is worth mentioning that two stage II CMC patients that were not included in the study were successfully treated for canine mucosal melanoma by us, before being treated for CMC. A combination of suicide and cytokine gene therapies had been applied, as described [14]. These long-term melanoma and mammary carcinoma survivors displayed total survivals >1766 and >2595 days, where the CMC survivals were >1448 and >1797, respectively. This fact supports the highly specific nature of the subcutaneous tumor vaccine that is the only difference between the two treatments, as well as the efficacy and safety of the treatment. Both patients are still alive and demonstrating a good quality of life.

Table 4 shows that most of the complete surgery treated patients (81%) were free of disease at the end of the study (this included the seven still alive patients), about 92% without local disease and about 88% without systemic disease (metastasis).

As discussed above, the initial stage of the disease had a strong influence on the outcome. There was a significant difference between the causes of death for the CS and PS patients. While only 31% of the complete surgery patients died because of CMC progression, 80% of the partial surgery patients did so (Table 4). This was a very encouraging outcome that supports further studies on this subject.

Preliminary data regarding a rescue scheme for the locally relapsing patients are shown in Table 5. A second local treatment of intratumoral lipoplexes carrying suicide and cIFNβ gene injections was offered to five patients (treated as described in Figure 1, but not included in the main trial, Table 1),to four when recurrence appeared after complete surgery and to one after a partial surgery. This action resulted in the following tumor control for the five treated patients: one complete response (CR), three partial responses (PR), and one stable disease (SD). Therefore, in the case of local recurrence or incomplete surgery, additional local gene therapy is feasible. More data are necessary to validate the efficacy this supplementary treatment.

The patients’ quality of life estimation was performed as described [14]. During the treatment, there was a high rate of re-establishment to the pre-disease situation of activity (72%), alert state (64%), appetite (72%), disposition (69%), and general welfare (75%).

The combined gene therapy treatment was safe, not allergenic, and could be applied numerous times. Rarely, some patients displayed minor adverse side effects that can be classified as grade ≤1 following the VCOG-CTCAE criteria [20]. Adverse effects included induration at the injection sites that resolved after 1–2 weeks (2/36, 5.5%), and a 1–2 day reduction of patient’s activity (5/36, 13.9%), after subcutaneous vaccination. The treatment did not produce any significant changes in the clinical and hematological parameters, local or systemic toxicity, and fever or organic dysfunction.

## 4. Conclusions

Based on our previous experience with canine melanoma [14], we proposed a similar scheme of this immunogene therapy approach for a new study in canine mammary carcinoma. As a surgery adjuvant, the treatment comprised a single post-surgical application of local cIFNβ plus suicide gene therapy, supplemented by the periodic subcutaneous allogeneic genetic vaccine. This vaccine was composed of whole tumor formolized extracts and lipoplexes carrying the hIL-2 and hGM-CSF genes.

After more than five years of follow up, we conclude that the proposed treatment applied after surgical excision of the tumor showed a high level of safety, even in long-term surviving patients that were periodically treated for many years.

It is interesting to compare our results with the already published data. Among the few published materials regarding the overall survival to CMC after the surgical removal of tumors, two studies provided valuable data. In the first article, stage II and III patients displayed median overall survivals of 579 and 252 days respectively [21]. In the second article stage II patients displayed a median overall survival of 406 days [22]. Beyond the actual differences in treatments made in different countries by different professionals at different times, it is worth noting that our treated canine patients displayed a median OS that largely exceeded such results, 788 days for stage II and 876 days for stage III.

As far as we know, the present trial is the largest veterinary cancer immunogene therapy study for canine mammary carcinoma reported to date. The high simplicity of the elements involved in this approach and the promising outcome reported here, encourage further trials in order to ascertain the efficacy of this approach, compared to a control group subjected to the best available approved treatment. In addition, a new larger study could provide a preclinical proof of concept and long term safety data for the eventual translation of a similar methodology to clinical trials for human breast cancer.

## Figures and Tables

**Table 1:** Patient demographics, disease stages at study entry, and survival times.

#	Breed	Initial Age (Years)	Stage	Surgery Type	Local Relapse	Cause of Death	Survival (Days)
1	Schnauzer	9	II	Complete	No	Alive	>306
2	Cocker spaniel	10	II	Complete	No	Alive	>491
3	Bichon frisé	12	II	Complete	No	Alive	>1498
4	Mixed breed	14	II	Complete	Yes	Unrelated	358
5	Cocker spaniel	13	II	Complete	No	Unrelated	422
6	Beagle	11	II	Complete	No	Unrelated	449
7	Mixed breed	13	II	Complete	No	Unrelated	496
8	Doberman pinscher	13	II	Complete	No	Discontinued	>580
9	Breton	13	II	Complete	No	Unrelated	788
10	Siberian husky	13	II	Complete	No	Discontinued	>1163
11	Mixed breed	12	II	Complete	No	Unrelated	1033
12	Mixed breed	9	III	Complete	No	Alive	>273
13	Pointer	11	III	Complete	No	Alive	>834
14	Cocker spaniel	12	III	Complete	No	Alive	>935
15	Boston terrier	13	III	Complete	No	Alive	>991
16	Cocker spaniel	13	III	Complete	Yes	Related	211
17	Mixed breed	10	III	Partial	* Yes	Related *	241
18	German shepherd	14	III	Partial	* Yes	Related	330
19	Mixed breed	11	III	Partial	* Yes	Related	352
20	Mixed breed	14	III	Complete	No	Discontinued	>384
21	Brittany	11	III	Complete	No	Discontinued	>575
22	Mixed breed	9	III	Complete	No	Unrelated	594
23	Cocker spaniel	12	III	Partial	* Yes	Related	708
24	Cocker spaniel	12	III	Complete	No	Discontinued	>775
25	Schnauzer	11	III	Complete	No	Unrelated	869
26	Rottweiler	13	III	Partial	* Yes	Unrelated	876
27	German shepherd	8	III	Partial	* Yes	Related	932
28	Mixed breed	7	III	Complete	No	Unrelated	1234
29	Cocker spaniel	10	III	Complete	No	Discontinued	>1345
30	German shepherd	11	IV	Complete	No	Related	99
31	Mixed breed	5	IV	Complete	No	Related	117
32	German shepherd	6	IV	Partial	* Yes	Related *	127
33	Mixed breed	15	IV	Partial	* Yes	Related *	170
34	Rottweiler	13	IV	Partial	* Yes	Unrelated	175
35	Mixed breed	11	IV	Complete	No	Related *	183
36	German shepherd	12	IV	Partial	* Yes	Related *	205

* PS patients were considered as relapsing at time 0 after surgery. Surgery: CS—complete; PS—partial.

**Table 2:** Median overall survivals obtained from Kaplan-Meier data (Figure 3a,b).

Survival	*n*	Overall (to Any Cause): OS	Overall (to CMC): MS	OS vs. MS
All stages	36	708 (99–498)	>1498 (99–1498)	NS
Stage II	11	788 (306–1498)	>1498 (306–1498)	NS
Stage III	18	876 (167–1345)	932(167–1345)	NS
Stage IV	7	170 (99–205)	170 (99–205)	NS
Stage II vs. Stage III		NS	*p* < 0.0423	**-**
Stage II vs. Stage IV		*p* < 0.0001	*p* < 0.0001	
Stage III vs. Stage IV		*p* < 0.0001	*p* < 0.0001	

Abbreviations: OS—overall survival to any cause; MS—overall survival to CMC; CMC—canine mammary carcinoma; NS—not significant. Patients were clinically evaluated and treated as described in Materials and Methods. *p*-values were calculated by Log-rank test for Kaplan–Meier analysis.

**Table 3:** Median overall and disease-free survivals obtained from Kaplan–Meier data (Figure 3c).

SURVIVAL (Days)	GT (*n =* 36)		CS vs. PS
	CS+GT (*n =* 26)	PS+GT (*n =* 10)	(*p*<)
Overall (any cause): OS	876 (99–498)	241 (127–932)	0.0006
Overall (to CMC): MS	>1498 (99–1498)	330 (127–932)	0.0001
	**Metastasis-free**	**Recurrence-free**	**Disease-free**
Survival (days)	>1498 (167–1498)	>1498 (99–1498)	>1498(99–1498)

Abbreviations: CS—complete surgery; GT—gene therapy combined treatment; NS—no significant; PS—partial surgery; DFS—disease-free survival; RFS—recurrence-free survival; MFS—metastasis-free survival. The patients were clinically evaluated and treated as described in Materials and Methods. The *p*-values were calculated by a Log-rank test for the Kaplan–Meier analysis. The stage IV patients starting with metastasis (*n =* 8) were not included in MFS and DFS, and those subjected to PS (*n =* 11) were not included in RFS and DFS calculations.

**Table 4:** Disease status at the end of the study and causes of death.

Patients	GT (*n =* 36)		
	CS+GT (*n =* 26)	PS+GT (*n =* 10)	CS+GT vs. PS+GT
	*n* (%)	*n* (%)	*p*<
Local disease-free	24 (92.3)	0 * (0)	0.0001
With local disease	2 (7.7)	10 * (100)	
Metastasis-free	23 (88.5)	6 (60.0)	NS
With metastasis	3 (11.5)	4 (40.0)	
Disease-free	21 (80.8)	0 * (0)	0.0001
With disease	5 (19.2)	10 (100)	
Alive	7 (26.9)	0 (0)	ND
Dead	13 (50.0)	10 (100)	
Dropped-out	6 (23.1)	0 (0)	
**Death**	*n* (%)	*n* (%)	*p*<
CMC-related	4 (30.8)	8 (80.0)	0.0361
CMC-unrelated	9 (69.2)	2 (20.0)	

Abbreviations: CS—complete surgery; GT—gene therapy combined treatment; ND—not determined; NS—not significant; PS—partial surgery. The patients were clinically evaluated and treated as described in Materials and Methods. *p*-values were calculated by 2-tail Fisher’s Exact test. * PS patients were considered as relapsing at time 0 after surgery.

**Table 5:** Outcome of an additional local treatment for local relapse.

#	Breed	Initial Age (Years)	Stage	Surgery Type	Local Relapse	Response to Additional Local GT	Cause of Death	Survival (Days)
1	Cocker spaniel	8	II	CS	Yes	PR	Alive	>2263
2	German shepherd	8	II	CS	Yes	PR	Related	325
3	Mixed breed	15	II	CS	Yes	SD	Related	365
4	Golden retriever	9	III	CS	Yes	CR	Unrelated	1263
5	Mixed breed	15	IV	PS	* Yes	PR	Related	167

* PS patients were considered as relapsing at time 0 after surgery. Responses: CR—complete; PR—partial; SD—stable disease. Surgery: CS—complete; PS—partial. Gene therapy—GT. After being treated as described in Figure 1, all of these patients (*p*) were subjected to additional cycles of GT (HSV*tk*/GCV and cIFNβ genes) when they had locally relapsed (p1–p4), or immediately after surgery (p5), as described in Materials and Methods.
